# Acute Kidney Injury in the Outpatient Setting Associates with Risk of End-Stage Renal Disease and Death in Patients with CKD

**DOI:** 10.1038/s41598-019-54227-6

**Published:** 2019-11-27

**Authors:** Hung-Chieh Yeh, I.-Wen Ting, Han-Chun Huang, Hsiu-Yin Chiang, Chin-Chi Kuo

**Affiliations:** 1 AKI-CARE (Clinical Advancement, Research and Education) Center, Department of Internal Medicine, China Medical University Hospital and College of Medicine, China Medical University, Taichung, Taiwan; 2 Division of Nephrology, Department of Internal Medicine, China Medical University Hospital and College of Medicine, China Medical University, Taichung, Taiwan; 3 Big Data Center, China Medical University Hospital and College of Medicine, China Medical University, Taichung, Taiwan; 4 School of Medicine, College of Medicine, China Medical University, Taichung, Taiwan

**Keywords:** Epidemiology, Acute kidney injury

## Abstract

Current acute kidney injury (AKI) diagnostic criteria are restricted to the inpatient setting. We proposed a new AKI diagnostic algorithm for the outpatient setting and evaluate whether outpatient AKI (AKI_OPT_) modifies the disease course among patients with chronic kidney disease (CKD) enrolled in the national predialysis registry. AKI_OPT_ was detected when a 50% increase in serum creatinine level or 35% decline in eGFR was observed in the 180-day period prior to enrollment in the predialysis care program. Outcomes were progression to end-stage renal disease (ESRD) and all-cause mortality. Association analyses were performed using multiple Cox regression and coarsened exact matching (CEM) analysis. Among 6,046 patients, 31.5% (1,905 patients) had developed AKI_OPT_ within the 180-day period before enrollment. The adjusted hazard ratios of the 1-year and overall risk of ESRD among patients with preceding AKI_OPT_ compared with those without AKI_OPT_ were 2.61 (95% CI: 2.15–3.18) and 1.97 (1.72–2.26), respectively. For 1-year and overall risk of all-cause mortality, patients with AKI_OPT_ had respectively a 141% (95% CI: 89–209%) and 84% (56–117%) higher risk than those without AKI_OPT_. This statistical inference remained robust in CEM analysis. We also discovered a complete reversal in the eGFR slope before and after the AKI_OPT_ from −10.61 ± 0.32 to 0.25 ± 0.30 mL/min/1.73 m^2^ per year; however, the loss of kidney function is not recovered. The new AKI_OPT_ diagnostic algorithm provides prognostic insight in patients with CKD.

## Introduction

By 2025, the International Society of Nephrology’s 0by25 initiative aims to eliminate all avoidable death by acute kidney injury (AKI) worldwide^1^. This goal seems to stem from advancements in medical big data and computing technology, which instantly allow for a large capacity of data collection and cloud storage. This capacity provides a solid foundation for real-time AKI monitoring and streamlined data management, particularly in hospital settings, to address unanswered questions regarding the nature and history of AKI^2^. However, research on health information technology reveals that the current burden of AKI may represent only part of a much larger problem in terms of the scale of community-acquired AKI (CA-AKI)^3^. Studies have demonstrated the mutually deteriorating interconnection between AKI and chronic kidney disease (CKD)^4,5^; thus, the clinical impacts of AKI are relatively complex and last in the long term, thereby characterizing the 0by25 initiative as overambitious.

The incidence of CA-AKI not requiring dialysis was first evaluated according to the criteria proposed by Hou *et al*. of hospital-acquired renal insufficiency using a database of 3.8 million individuals from Kaiser Permanente of Northern California, and it was estimated at 384.1 per 100,000 person-years between 1996 and 2003^6,7^. In the United Kingdom, the incidence of CA-AKI was estimated to be 6.4% in the catchment area of Southeast Wales from 2011–2012 using creatinine criteria of the AKI Network classification^8,9^. In 2013, the first large-scale estimate of CA-AKI prevalence in the Chinese population was conducted at 44 hospitals of 22 provinces in four geographic regions of China with 2.2 million adult patients based on the 2012 KDIGO definition of AKI^10,11^. The detection rate of CA-AKI was 2.03%, and the most notable finding was that only 25% of CA-AKI cases were identified by supervising clinicians, indicating a critical health care gap in AKI^1,10^. In Taiwan, the estimated incidence of CA-AKI without preexisting CKD in a retrospective single-center cohort of 395,219 patients was 1.68% between 2010 and 2014 according to the Risk, Injury, Failure, Loss of Kidney Function, and End-Stage Kidney Disease (RIFLE) classification^12,13^. However, all of the aforementioned large epidemiologic studies were conducted in hospitalized populations.

To narrow this research gap, we evaluated the prognostic role of fluctuation in kidney function measured according to serum creatinine or estimated glomerular filtration rate (eGFR) in the outpatient setting throughout the 180-day period before the CKD patients were enrolled in a national pre-end-stage renal disease (pre-ESRD) care program. This phenotype of AKI was identified using our proposed diagnostic algorithm in an outpatient setting and was named outpatient AKI (AKI_OPT_). To avoid confusion, the term of CA-AKI is used for AKI that is speculated to occur outside the hospital according to the peak serum creatinine measured specifically in the hospital. By contrast, the diagnosis of AKI_OPT_ is based on all available serum creatinine levels prior to the outpatient service no matter they were measured in the outpatient or inpatient setting.

## Results

The study cohort was composed of a total of 6,046 patients enrolled in the pre-ESRD program, contributing to a total of 13,467.68 person-years of follow-up. The median age at enrollment in the pre-ESRD program was 67. 4 years (IQR: 56.9–76.5 years). The median follow-up times for outcomes of ESRD requiring dialysis and all-cause mortality were 1.68 (IQR: 0.80–3.01) and 1.69 (IQR: 0.81–3.03) years, respectively. Overall, 68.5% (4,141 patients) of the study population did not meet the diagnostic threshold of AKI_OPT_, whereas the remaining 31.5% (1,905 patients) had developed AKI_OPT_. Among patients with CKD who had a history of AKI_OPT_, 80.7% (n = 1573) had stable AKI_OPT_ and nearly 20% (n = 368) had deteriorating AKI_OPT_ (Table 1). Both maximum and minimum serum creatinine level were measured in the inpatient setting for 5% of the study population.

**Table 1:** Baseline demographic and clinical characteristics of the study population by the presence of preceding AKI_OPT_.

Variables	No AKI_OPT_	AKI_OPT_	AKI_OPT_ status		*p*-value	
			Stable AKI_OPT_	Deteriorating AKI_OPT_	No AKI_OPT VS_ AKI_OPT_	No AKI_OPT VS_ AKI_OPT_ status
Participant, n	4141	1905	1537	368	─	─
Proportion of the study population (%)	68.5	31.5	25.4	6.1	─	─
Proportion of patients with AKI_OPT_ (%)	─	─	80.7	19.3	─	─
Age at entry (year)	66.7 (56.4, 76.0)	68.9 (58.7, 77.3)	69.6 (59.2, 78.1)	65.5 (56.3, 74.8)	<0.001	<0.001
Body mass index	24.4 (22.1, 27.2)	23.8 (21.3, 26.8)	23.9 (21.3, 26.8)	23.4 (21.1, 27.0)	<0.001	<0.001
Time from the lowest eGFR to pre-ESRD enrollment (month)	0.10 (0.00, 1.74)	0.43 (0.00, 1.67)	0.52 (0.00, 1.84)	0.00 (0.00, 0.62)	<0.001	<0.001
Serum creatinine variability (mg/dL) – 180 days prior to pre-ESRD enrollment						
Minimum	1.76 (1.27, 3.10)	1.80 (1.21, 3.04)	1.69 (1.18, 2.66)	2.87 (1.64, 4.60)	0.241	<0.001
Maximum	2.08 (1.49, 3.70)	3.64 (2.40, 6.30)	3.41 (2.30, 5.47)	5.89 (3.18, 8.85)	<0.001	<0.001
Difference^1^	0.30 (0.16, 0.60)	1.63 (0.98, 3.00)	1.51 (0.93, 2.66)	2.57 (1.31, 4.20)	<0.001	<0.001
Percent change (%)^2^	17.2 (9.8, 27.0)	77.1 (54.9, 122.0)	77.8 (55.2, 121.8)	76.3 (53.7, 122.0)	<0.001	<0.001
eGFR variability (ml/min/1.73m^2^) – 180 days prior to pre-ESRD enrollment						
Minimum	29.1 (14.2, 45.0)	13.7 (7.6, 23.8)	15.1 (8.7, 25.1)	8.1 (5.2, 16.4)	<0.001	<0.001
Maximum	35.7 (17.6, 54.4)	32.5 (18.0, 54.1)	35.6 (21.0, 56.8)	19.3 (11.0, 37.9)	0.685	<0.001
Difference^3^	5.0 (2.4, 9.2)	16.1 (8.6, 28.4)	18.2 (10.2, 30.1)	10.1 (5.4, 21.0)	<0.001	<0.001
Percent change (%)^4^	17.0 (10.0, 24.8)	49.6 (40.9, 61.5)	49.6 (40.9, 61.5)	49.6 (40.4, 61.6)	<0.001	<0.001
Woman, n (%)	1710 (41.29)	887 (46.56)	714 (46.45)	173 (47.01)	<0.001	0.001
Smoking, n (%)					0.046	0.024
Never	3435 (82.95)	1610 (84.51)	1300 (84.58)	310 (84.24)		
Former	303 (7.32)	147 (7.72)	126 (8.20)	21 (5.71)		
Current	403 (9.73)	148 (7.77)	111 (7.22)	37 (10.05)		
Alcohol consumption, n (%)					0.008	0.008
Never	3787 (91.45)	1747 (91.71)	1403 (91.28)	344 (93.48)		
Former	214 (5.17)	118 (6.19)	103 (6.70)	15 (4.08)		
Current	140 (3.38)	40 (2.10)	31 (2.02)	9 (2.45)		
Education, n (%)					<0.001	<0.001
<9 yrs	1066 (25.74)	615 (32.28)	499 (32.47)	116 (31.52)		
9 ≤ ~<12 yrs	1595 (38.52)	758 (39.79)	608 (39.56)	150 (40.76)		
12 ≤ ~<16 yrs	968 (23.38)	394 (20.68)	315 (20.49)	79 (21.47)		
16 + yrs	512 (12.36)	138 (7.24)	115 (7.48)	23 (6.25)		
Primary etiologies of CKD, n (%)					<0.001	<0.001
CGN	1557 (37.70)	584 (30.69)	479 (31.18)	105 (28.61)		
Systemic disease	2278 (55.16)	1176 (61.80)	943 (61.39)	233 (63.49)		
Obstructive nephropathy	95 (2.30)	77 (4.05)	64 (4.17)	13 (3.54)		
Other nephropathy	200 (4.84)	66 (3.47)	50 (3.26)	16 (4.36)		
CKD stage at enrollment, n (%)					<0.001	<0.001
1	173 (4.18)	18 (0.94)	18 (1.17)	0 (0.00)		
2	315 (7.61)	55 (2.89)	53 (3.45)	2 (0.54)		
3	1779 (42.96)	572 (30.03)	521 (33.90)	51 (13.86)		
4	949 (22.92)	625 (32.81)	545 (35.46)	80 (21.74)		
5	925 (22.34)	635 (33.33)	400 (26.02)	235 (63.86)		
Diabetes, n (%)	1608 (38.84)	1019 (53.52)	833 (54.23)	186 (50.54)	<0.001	<0.001
Hypertension, n (%)	2482 (59.95)	1294 (67.96)	1043 (67.90)	251 (68.21)	<0.001	<0.001
Cardiovascular disease, n (%)	1595 (38.53)	922 (48.48)	771 (50.26)	151 (41.03)	<0.001	<0.001
Medication utilization 90 days prior to AKI_OPT_, n (%)						
NSAIDs	476 (11.51)	392 (20.60)	342 (22.28)	50 (13.59)	<0.001	<0.001
Contrast	217 (5.25)	309 (16.24)	263 (17.13)	46 (12.50)	<0.001	<0.001
ACEI	623 (15.06)	329 (17.29)	269 (17.52)	60 (16.30)	0.028	0.075
ARBs	1538 (37.19)	676 (35.52)	543 (35.37)	133 (36.14)	0.213	0.443
Diuretics	1415 (34.21)	1062 (55.81)	830 (54.07)	232 (63.04)	<0.001	<0.001
Medication utilization one year prior to pre-ESRD enrollment, n (%)						
Pentoxifylline	990 (23.94)	434 (22.81)	357 (23.26)	77 (20.92)	0.337	0.402
NSAIDs	1053 (25.46)	728 (38.26)	620 (40.39)	108 (29.35)	<0.001	<0.001
Contrast	588 (14.22)	618 (32.48)	518 (33.75)	100 (27.17)	<0.001	<0.001
*Anti-platelet*						
Aspirin	1193 (28.84)	684 (35.94)	570 (37.13)	114 (30.98)	<0.001	<0.001
Dipyridamole	304 (7.35)	132 (6.94)	106 (6.91)	26 (7.07)	0.564	0.842
other Anti-platelet agents	392 (9.48)	333 (17.50)	280 (18.24)	53 (14.40)	<0.001	<0.001
*Urate-lowering/gout related medications*						
Allopurinol	516 (12.48)	241 (12.66)	189 (12.31)	52 (14.13)	0.837	0.626
Febuxostat	68 (1.64)	74 (3.89)	56 (3.65)	18 (4.89)	<0.001	<0.001
Benzbromarone	508 (12.28)	199 (10.46)	169 (11.01)	30 (8.15)	0.040	0.038
Colchicine	504 (12.19)	294 (15.45)	234 (15.24)	60 (16.30)	0.001	0.002
Sulfinpyrazone	50 (1.21)	24 (1.26)	22 (1.43)	2 (0.54)	0.864	0.373
*Anti-hypertensive agents*						
ACEI	970 (23.45)	586 (30.79)	482 (31.40)	104 (28.26)	<0.001	<0.001
ARBs	1896 (45.84)	932 (48.98)	756 (49.25)	176 (47.83)	0.023	0.068
Diuretics	1875 (45.33)	1374 (72.20)	1096 (71.40)	278 (75.54)	<0.001	<0.001
*Anti-diabetic agents*						
Oral hypoglycemic agents	1332 (32.21)	781 (41.04)	640 (41.69)	141 (38.32)	<0.001	<0.001
Insulin	796 (19.25)	858 (45.09)	710 (46.25)	148 (40.22)	<0.001	<0.001
Baseline biochemical parameters						
eGFR (mL/min/1.73m^2^)	30.8 (15.1, 47.6)	20.1 (10.7, 33.1)	22.9 (13.1, 36.0)	9.5 (5.6, 18.5)	<0.001	<0.001
Serum creatinine (mg/dL)	1.90 (1.37, 3.47)	2.62 (1.69, 4.53)	2.40 (1.61, 3.92)	4.43 (2.45, 7.71)	<0.001	<0.001
Blood urea nitrogen (mg/dL)	30.0 (19.0, 49.0)	40.0 (25.0, 63.0)	38.0 (24.0, 58.0)	59.0 (35.0, 85.0)	<0.001	<0.001
Serum uric acid (mg/dL)	7.30 (6.10, 8.60)	7.70 (6.40, 9.30)	7.60 (6.30, 9.30)	7.90 (6.60, 9.30)	<0.001	<0.001
Calcium (mg/dL)	8.90 (8.40, 9.20)	8.70 (8.20, 9.10)	8.70 (8.30, 9.10)	8.65 (8.10, 9.05)	<0.001	<0.001
Phosphate (mg/dL)	4.10 (3.60, 4.80)	4.30 (3.60, 5.20)	4.20 (3.60, 5.00)	4.70 (4.00, 6.00)	<0.001	<0.001
Serum albumin (g/dL)	3.90 (3.40, 4.20)	3.60 (3.10, 4.00)	3.60 (3.10, 4.00)	3.50 (3.00, 4.00)	<0.001	<0.001
Hemoglobin (g/dL)	11.2 (9.5, 13.1)	10.2 (9.0, 11.8)	10.3 (9.2, 11.9)	9.7 (8.5, 11.0)	<0.001	<0.001
Urine PCR (mg/g)	781 (201, 2442)	1293 (290, 3916)	1080 (251, 3483)	1970 (741, 5911)	<0.001	<0.001

^1^Difference of serum creatinine = maximum serum creatinine -minimum serum creatinine.

^2^Percent change of serum creatinine = (maximum serum creatinine -minimum serum creatinine)/minimum serum creatinine × 100%.

^3^Difference of eGFR = maximum eGFR -minimum eGFR.

^4^Percent change of eGFR = (maximum eGFR -minimum eGFR)/maximum eGFR × 100%.

*p*-values are calculated by Wilcoxon rank sum test for continuous variables and Chi-square test for categorical variables.

**Abbreviations:** ACEI: angiotensin-converting enzyme inhibitor, AKI: acute kidney injury, ARB: angiotensin II receptor blocker, CGN: chronic glomerulonephritis, CKD: chronic kidney disease, eGFR: estimated glomerular filtration rate, IQR: inter-quartile range, NSAID: non-steroidal anti-inflammatory drug, PCR: protein to creatinine ratio.

Compared with patients without AKI_OPT_, those with AKI_OPT_ episodes tended to be older, female, nonsmokers, less educated, and have lower BMI and etiologies related to systemic diseases (e.g., diabetes, hypertension, and CVD) (Table 1). In addition to medication use for comorbidities that commonly accompany AKI_OPT_, exposure to nephrotoxic agents such as NSIADs and radiocontrast was more prevalent among CKD patients with AKI_OPT_. Patients with a history of AKI_OPT_ were also more likely to receive hypouricemic and antigout therapy than were those without AKI_OPT_ (Table 1). At the time of enrollment in the pre-ESRD program, patients with deteriorating CA-AKI had the lowest median eGFR (9.5 vs. 30.8 mL/min/1.73 m^2^ in patients without AKI_OPT_ and 22.9 mL/min/1.73 m^2^ in patients with stable AKI_OPT_). The median difference and percent change between the maximum and minimum serum creatinine levels were 0.30 mg/dL (IQR: 0.16–0.60) and 17.2% (IQR: 9.8–27.0) and 1.63 mg/dL (IQR: 0.98–3.00) and 77.1% (IQR: 54.9–122.0), respectively, for patients without and with AKI_OPT_. Kidney function markers, including serum creatinine, blood urea nitrogen, and urine protein to creatinine ratio, demonstrated a significant increasing trend across the AKI_OPT_ subgroups (No AKI_OPT_, stable AKI_OPT_, and deteriorating AKI_OPT_). For hemoglobin and serum albumin, corresponding decreasing trends were observed (Table 1).

**Figure 1:**
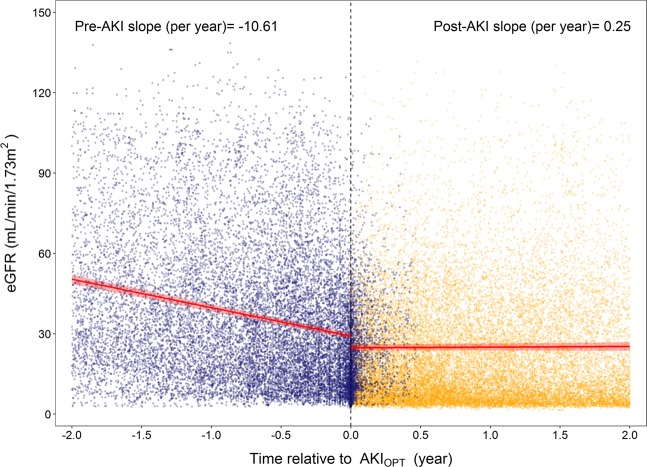
eGFR slope (red line) with the light red shaded area representing 95% confidence intervals before and after the AKI_OPT_ event, modeled using the growth piecewise linear mixed model by incorporating random effects. Blue and orange points represent eGFR measurements before and after enrollment into pre-ESRD program.

**Figure 2:**
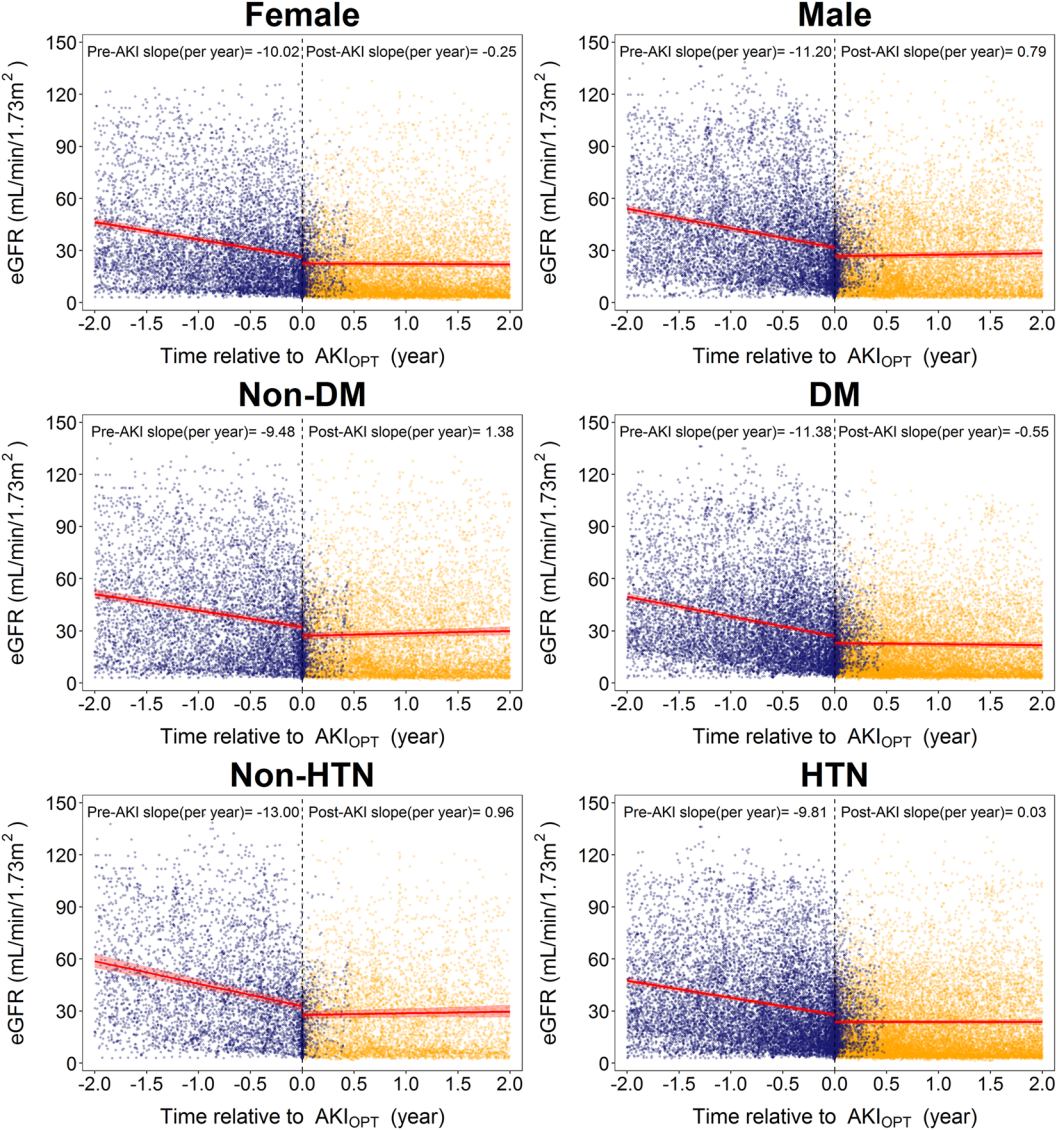
eGFR slope (red line) with the light red shaded area representing 95% confidence intervals before and after the AKI_OPT_ event, stratified by sex and the comorbidities of diabetes and hypertension. Blue and orange points represent eGFR measurements before and after enrollment into pre-ESRD program.

In multiple Cox regression analyses, the fully adjusted hazard ratios (aHRs) of the 1-year and overall risk of ESRD among patients with AKI_OPT_ were 2.61 (95% CI: 2.15–3.18) and 1.97 (95% CI: 1.72–2.26), respectively, compared with those without a history of AKI_OPT_ (Table 2**, Model 4, ESRD**). Among patients with deteriorating AKI_OPT_, the 1-year and overall risk of ESRD increased by 272% (95% CI: 175%–402%) and 152% (95% CI: 98%–221%), respectively. The corresponding risk differences were 115% (95% CI: 75–164%) and 77% (95% 53–104%) among patients with stable AKI_OPT_ (Table 2**, Model 4, ESRD**). For 1-year and overall risk of all-cause mortality, patients with AKI_OPT_ had respectively a 141% (95% CI: 89%–209%) and 84% (95% CI: 56%–117%) higher risk than those without AKI_OPT_ (Table 2**, Model 4, all-cause mortality**). Patients with stable and deteriorating AKI_OPT_ had hazard ratios for 1-year and overall all-cause mortality of 2.41 (95% CI: 1.86–3.13) and 1.79 (95% CI: 1.50–2.13), and 2.41 (95% CI: 1.57–3.70) and 2.07 (95% CI: 1.52–2.81), respectively (Table 2**, Model 4, all-cause mortality**). The growth piecewise linear mixed modeling revealed a complete reversal in the eGFR slope before and after the AKI_OPT_ event from −10.61 ± 0.32 to 0.25 ± 0.30 mL/min/1.73 m^2^ per year (Fig. 1. However, the loss of kidney function could not be recovered after a 2-year follow-up. Among patients with diabetes and renin-angiotensin system (RAS) inhibitors, the post- AKI_OPT_ slope remained negative at −0.55 ± −0.39 and −0.39 ± 0.40 mL/min/1.73 m^2^ per year, respectively (Fig. 2, Table 3, and Supplementary Fig. S1 and Table S1).

**Table 2:** Hazard ratios (95% confidence interval) for risk of progression to ESRD and all-cause mortality by the presence of preceding AKI_OPT_.

	Case/N	Person-years	Incidence	Crude HR (95% CI)	Model 1	Model 2	Model 3	Model 4
					Adjusted HR (95% CI)	Adjusted HR (95% CI)	Adjusted HR (95% CI)	Adjusted HR (95% CI)
**ESRD requiring dialysis**†								
**1-year dialysis**^**a**^								
No AKI_OPT_	321/4141	3629.92	88.43	1.00 (Ref)	1.00 (Ref)	1.00 (Ref)	1.00 (Ref)	1.00 (Ref)
AKI_OPT_	286/1905	1455.19	196.54	2.11 (1.80, 2.48)	2.14 (1.83, 2.52)	2.00 (1.70, 2.36)	1.78 (1.50, 2.12)	2.61 (2.15, 3.18)
No AKI_OPT_	321/4141	3629.92	88.43	1.00 (Ref)	1.00 (Ref)	1.00 (Ref)	1.00 (Ref)	1.00 (Ref)
Stable AKI_OPT_	176/1537	1226.01	143.56	1.55 (1.29, 1.87)	1.59 (1.32, 1.91)	1.47 (1.22, 1.78)	1.33 (1.09, 1.62)	2.15 (1.75, 2.64)
Deteriorating AKI_OPT_	110/368	229.18	479.97	4.98 (3.99, 6.21)	4.82 (3.85, 6.04)	4.54 (3.61, 5.71)	3.71 (2.93, 4.69)	3.72 (2.75, 5.02)
*P for trend*				<*0.001*	<*0.001*	<*0.001*	<*0.001*	<*0.001*
**Overall**								
No AKI_OPT_	753/4141	10298.25	73.12	1.00 (Ref)	1.00 (Ref)	1.00 (Ref)	1.00 (Ref)	1.00 (Ref)
AKI_OPT_	460/1905	3169.43	145.14	1.67 (1.48, 1.87)	1.69 (1.50, 1.90)	1.59 (1.41, 1.80)	1.50 (1.32, 1.71)	1.97 (1.72, 2.26)
No AKI_OPT_	753/4141	10298.25	73.12	1.00 (Ref)	1.00 (Ref)	1.00 (Ref)	1.00 (Ref)	1.00 (Ref)
Stable AKI_OPT_	314/1537	2685.45	116.93	1.35 (1.19, 1.54)	1.39 (1.22, 1.58)	1.30 (1.13, 1.49)	1.24 (1.07, 1.43)	1.77 (1.53, 2.04)
Deteriorating AKI_OPT_	146/368	483.98	301.67	3.34 (2.76, 4.05)	3.19 (2.62, 3.89)	3.05 (2.49, 3.73)	2.68 (2.17, 3.31)	2.52 (1.98, 3.21)
*P for trend*				<*0.001*	<*0.001*	<*0.001*	<*0.001*	<*0.001*
**All-cause mortality**								
**1-year mortality**^**b**^								
No AKI_OPT_	131/4141	3635.61	36.03	1.00 (Ref)	1.00 (Ref)	1.00 (Ref)	1.00 (Ref)	1.00 (Ref)
AKI_OPT_	156/1905	1460.99	106.78	2.97 (2.35, 3.74)	2.75 (2.18, 3.48)	2.69 (2.12, 3.42)	2.20 (1.73, 2.82)	2.41 (1.89, 3.09)
No AKI_OPT_	131/4141	3635.61	36.03	1.00 (Ref)	1.00 (Ref)	1.00 (Ref)	1.00 (Ref)	1.00 (Ref)
Stable AKI_OPT_	130/1537	1228.83	105.79	2.94 (2.31, 3.75)	2.70 (2.11, 3.44)	2.63 (2.05, 3.38)	2.14 (1.66, 2.77)	2.41 (1.86, 3.13)
Deteriorating AKI_OPT_	26/368	232.16	111.99	3.12 (2.05, 4.76)	3.07 (2.01, 4.68)	3.03 (1.98, 4.63)	2.56 (1.67, 3.92)	2.41 (1.57, 3.70)
*P for trend*				<*0.001*	<*0.001*	<*0.001*	<*0.001*	<*0.001*
**Overall**								
No AKI_OPT_	377/4141	10331.20	36.49	1.00 (Ref)	1.00 (Ref)	1.00 (Ref)	1.00 (Ref)	1.00 (Ref)
AKI_OPT_	287/1905	3198.17	89.74	2.43 (2.08, 2.84)	2.15 (1.84, 2.52)	2.04 (1.74, 2.39)	1.74 (1.48, 2.06)	1.84 (1.56, 2.17)
No AKI_OPT_	377/4141	10331.20	36.49	1.00 (Ref)	1.00 (Ref)	1.00 (Ref)	1.00 (Ref)	1.00 (Ref)
Stable AKI_OPT_	238/1537	2701.02	88.11	2.39 (2.03, 2.82)	2.07 (1.76, 2.44)	1.95 (1.65, 2.31)	1.66 (1.40, 1.98)	1.79 (1.50, 2.13)
Deteriorating AKI_OPT_	49/368	497.15	98.56	2.66 (1.98, 3.59)	2.65 (1.96, 3.58)	2.58 (1.91, 3.49)	2.23 (1.64, 3.02)	2.07 (1.52, 2.81)
*P for trend*				<*0.001*	<*0.001*	<*0.001*	<*0.001*	<*0.001*

^†^With competing risk analysis for death.

^a^1-year dialysis: ESRD requiring dialysis within 1 year following pre-ESRD enrollment.

^b^1-year mortality: All-cause mortality within 1 year following pre-ESRD enrollment.

Incidence = No. of incident dialysis cases/person-years*1000.

Model 1: Adjusted for age at entry, gender, smoking status, alcohol consumption, education (n = 6046).

Model 2: Further adjusted for diabetes, hypertension, cardiovascular disease, and primary etiologies of CKD (n = 6029).

Model 3: Adjusted for medication utilization within 90 days prior to AKI_OPT_ (n = 6024).

Model 4: Adjusted for the baseline serum creatinine (n = 6024).

**Abbreviations:** AKI_OPT_: acute kidney injury in outpatient setting, CI: confidence interval, CKD: chronic kidney disease, ESRD: end-stage renal disease, HR: hazard ratio.

**Table 3:** Estimates of the main fixed effects obtained from the growth piecewise mixed-effects modeling.

	Growth piecewise mixed-effects model			
	Estimate ± SE (ml/min/1.73 m^2^)	*p*-value	Estimate ± SE (ml/min/1.73 m^2^)	*p* -value
**Overall**				
Intercept	24.75 ± 0.59	<0.001	─	─
Pre-AKI_OPT_ slope (yr^−1^)	−10.61 ± 0.32	<0.001	─	─
Post-AKI_OPT_ slope (yr^−1^)	0.25 ± 0.30	0.420	─	─
	**Woman**		**Man**	
Intercept	22.48 ± 0.83	<0.001	26.79 ± 0.82	<0.001
Pre-AKI_OPT_ slope (yr^−1^)	−10.02 ± 0.42	<0.001	−11.20 ± 0.48	<0.001
Post-AKI_OPT_ slope (yr^−1^)	−0.25 ± 0.39	0.534	0.79 ± 0.46	0.082
	**Non-Diabetes**		**Diabetes**	
Intercept	27.26 ± 1.01	<0.001	22.95 ± 0.69	<0.001
Pre-AKI_OPT_ slope (yr^−1^)	−9.48 ± 0.52	<0.001	−11.38 ± 0.41	<0.001
Post-AKI_OPT_ slope (yr^−1^)	1.38 ± 0.48	0.004	−0.55 ± 0.39	0.157
	**Non-Hypertension**		**Hypertension**	
Intercept	27.77 ± 1.25	<0.001	23.69 ± 0.66	<0.001
Pre-AKI_OPT_ slope (yr^−1^)	−13.00 ± 0.82	<0.001	−9.81 ± 0.33	<0.001
Post-AKI_OPT_ slope (yr^−1^)	0.96 ± 0.78	0.219	0.03 ± 0.31	0.915

Linear model: *eGFR*_*ij*_ = *β*_0_ + *β*_1_(*Age*_*ij*_ − *Age*_*at AKI*_) × *δ*_*ij*_ + *β*_2_(*Age*_*ij*_ − *Age*_*at AKI*_) × (1 − *δ*_*ij*_) + *ε*_*ij*_.

*δ*_*ij*_ = 1, for the time period before AKI event; *δ*_*ij*_ = 0, for the time period after AKI event.

**Abbreviations:** AKI_OPT_, acute kidney injury in outpatient setting; SE, standard error.

**Figure 3:**
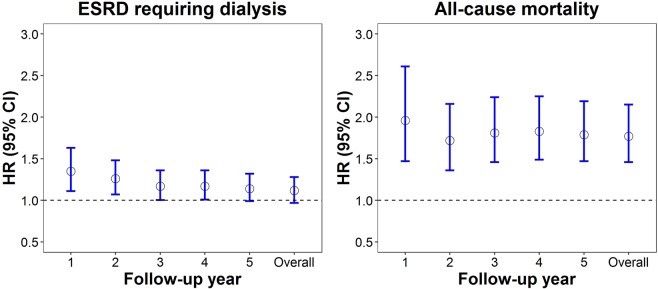
Hazard ratios (95% confidence interval) for risks of progression to ESRD and all-cause mortality using coarsened exact matching analysis for 1-, 2-, 3-, 4-, and 5-year and overall follow-up period comparing patients with AKI_OPT_ versus non-AKI_OPT_ before pre-dialysis program enrollment.

**Figure 4:**
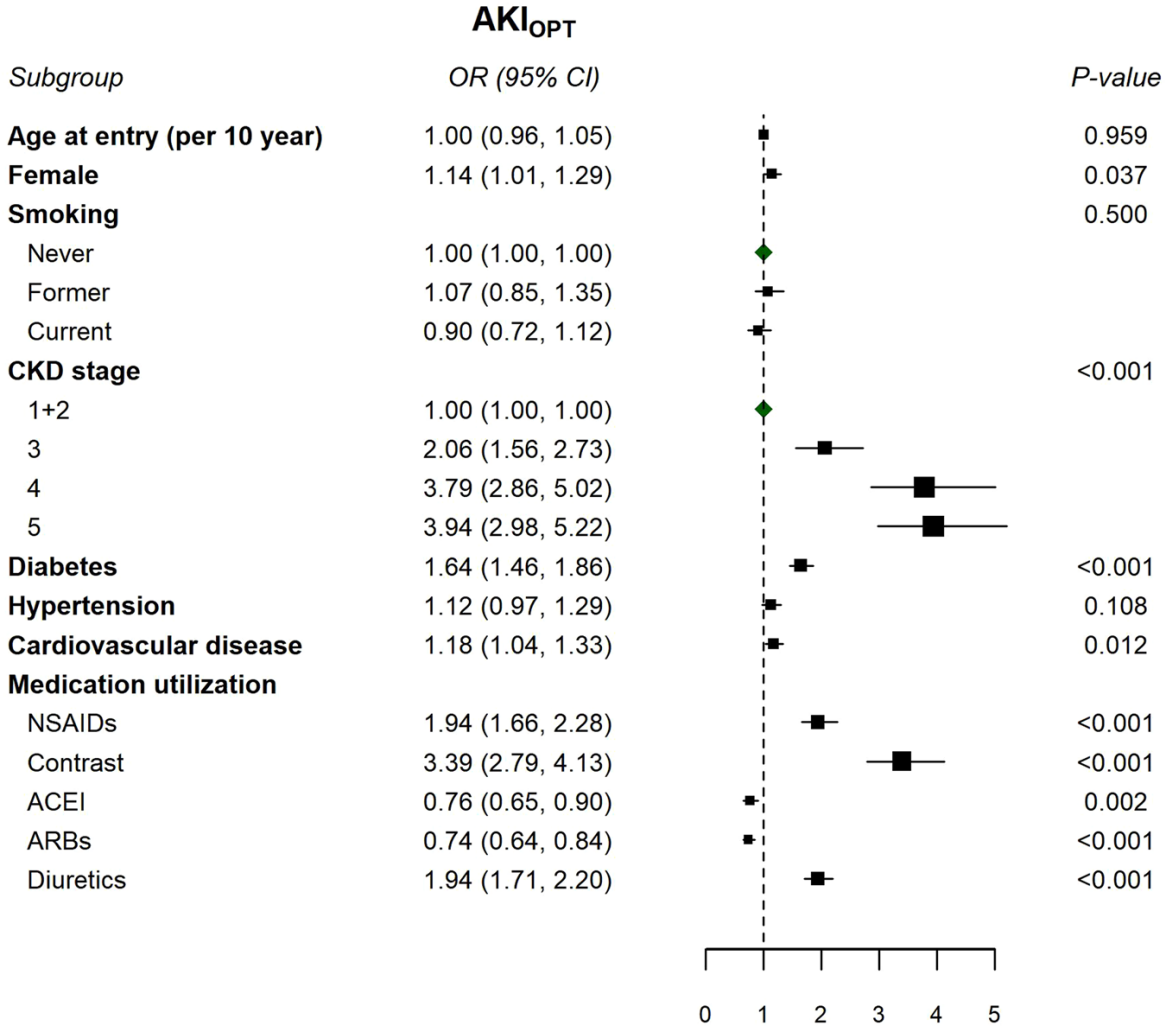
Cross-sectional associations between the demographic and clinical factors and the AKI_OPT_ status, illustrated in a multivariable logistic regression model. **Abbreviations:** AKI_OPT_, acute kidney injury in outpatient setting; CI, confidence interval; OR, odds ratio.

CEM analysis revealed that the effects of AKI_OPT_ on the progression to ESRD gradually attenuated in subsequent years (e.g., aHR [1.44, 95% CI: 1.10–1.77] for 1-year mortality to aHR [1.10, 95% CI: 0.99–1.41] for 5-year mortality) following pre-ESRD enrollment; however, its effects on all-cause mortality were stable, ranging from an aHR of 1.7 to 1.9 throughout the follow-up period (Fig. 3**)**. Supplementary Table S2 indicates that the matched variables in CEM between patients with and without AKI_OPT_ were well balanced. In the multiple logistic regression of risk markers associated with the risk of developing AKI_OPT_, we found female gender, advanced CKD stage, diabetes, CVD, and the utilization of NSAIDs, contrast, and diuretics were significantly associated with AKI_OPT_ (Fig. 4).

## Discussion

The history of acute change in kidney function prior to pre-ERSD enrollment is prognostically critical in risk assessment and management in patients with CKD. In the present study, patients with AKI_OPT_ were associated with a higher risk of progression to ESRD and all-cause mortality than were those without a history of AKI_OPT_. The risk was particularly high among patients with the deteriorating type of AKI_OPT_. We also found that the loss of kidney function before and during the AKI_OPT_ event could not be completely recovered even with meticulous multidisciplinary care. The study results not only provide insight into how AKI_OPT_ modifies the course of CKD but also emphasize the unmet need for the development of a universal screening-based diagnostic workflow to detect AKI_OPT_.

The first systematic evaluation of CA-AKI conducted by Kaufman in 1991 established the basic concept of CA-AKI detection^14^. The main diagnostic scheme is to screen admitted patients for impaired kidney function and then track the history, or the baseline serum creatinine level (the lowest reference serum creatinine level) if available, within the 12 months prior to admission or prospective serum creatinine during the entire hospital course^14^. Before the RIFLE criteria for AKI was proposed in 2004, all research had defined CA-AKI passively in the inpatient setting using Kaufman’s approach^6,15–18^. For instance, Obialo *et al*. defined CA-AKI as serum creatinine levels of >2.0 mg/dL at admission without a history of kidney disease^17^. Similarly, Hsu *et al*. defined the incidence of CA-AKI in a large community-based population according to all available inpatient serum creatinine measurements and then selected reference and index serum creatinine level to estimate the incidence of AKI using diagnostic criteria proposed by Hou *et al*.^6,7^. After 2010, researchers began to use RIFLE-based criteria to define CA-AKI in the inpatient setting^8,19,20^, except for Talabani *et al*., who used a fixed cohort covering the outpatient setting^21^. Collectively, a clear trend was observed and indicated that CA-AKI is more prevalent than HA-AKI and has a much lower mortality rate^22^. However, it remains unclear why compared with patients with HA-AKI, patients with CA-AKI tend to be classified in the highest AKI severity group but have a much lower risk of mortality^22^. This observation questions the feasibility of applying RIFLE-based inpatient AKI criteria for patients from the community.

The diagnostic algorithm we proposed changes the paradigm for AKI diagnosis and extends the scope of clinical AKI into the outpatient setting. Based on longitudinal clinical data, we verified our proposed definition; a 50% fluctuation in serum creatinine in the 180-day period prior to the pre-ESRD enrollment significantly modified the course of CKD. More importantly, we discovered that complete recovery of kidney function after AKI_OPT_ is unlikely in patients with CKD. The change in eGFR slope from −10.61 back to 0.25 mL/min/1.73 m^2^ represents the acute and reversible nature of kidney injury as the accelerated rate in deterioration of kidney function can be recovered; however, loss of kidney function could not be completely regained as the post-AKI eGFR slope was not steep upward enough to recover to the baseline kidney function before the event of AKI_OPT_ (Fig. 1). This finding is in concordance with prior evidence indicating that HA-AKI defined using RIFLE-based criteria increases the risk of de novo CKD and accelerates CKD progression in critically ill patients^23–25^ and admitted patients^26,27^ and that a dose–response relationship exists between AKI stage and CKD progression^28^. However, these inferences are limited to inpatient settings and underestimate the true effect of AKI, particularly CA-AKI, which has yet to be adequately defined in the literature.

In the present study, when we applied the KDIGO AKI criteria, only 2,758 patients exhibited consecutive serum creatinine measurements within a 7-day time period. Among them, 699 patients had AKI, and 16.3% of the AKI patients exhibited peak serum creatinine levels in inpatient settings (Data not shown). Therefore, it is not feasible to use RIFLE-based diagnostic criteria to detect AKI in the community. Indeed, the difference between AKI_OPT_ and rapid progressive CKD (when annual eGFR declining rate >5 mL/min/1.73 m^2^) may be marginal as the two phenotypes shared common risk factors such as nephrotoxic agents, dehydration, or obstructive uropathy^29,30^. It is therefore difficult to differentiate AKI_OPT_ from so called rapid progression of CKD, particularly at the onset of these events. Furthermore, if triggering factors of acute kidney insults are not promptly identified and managed, the reversibility of the AKI and recovery of kidney function will then develop into an irreversible kidney injury leading to the phenotype of rapid progression of CKD with an annual eGFR declining rate persistently >5 mL/min/1.73 m^2^. However, we found a significantly slower progression (from approximately −10 mL/min/1.73 m^2^ per year to no progression) after AKI events, which contradicts traditional notions of persistent chronicity. This observation signifies that the phenotype of AKI_OPT_ identified by our proposed criteria is clearly different from that of rapid progression of CKD. The mechanisms underlying the AKI-CKD continuum have been extensively explored in animal models^5^. Maladaptive repair, infiltration of inflammatory cells, stimulation of fibrocysts and myofibrocysts, and tubulointersitital fibrosis have been linked to the development of de novo CKD and CKD progression after AKI^31^. These injurious molecular pathways are triggered in intrarenal microenvironments rich in damage-associated molecular patterns that are sustained by mutually aggravating mechanisms such as hypoxia, reactive oxygen species, or inflammation^32,33^. These mechanistic insights provide conceptual coherence between laboratory and epidemiological findings that supports the causality of AKI-to-CKD progression.

The increased risk of progression to ESRD gradually decreased within 5 years following the pre-ESRD enrollment with the highest risk appearing in the first year (Fig. 3). This finding can be explained by the sudden drop of eGFR before the AKI_OPT_ event and the slow increase of eGFR after the AKI_OPT_ event (Fig. 1). The significant loss of kidney function before AKI_OPT_ event may put patients in advanced CKD stage, which increases their risk of progression to ESRD in the first two years following the event of AKI_OPT_ because regain of kidney function is unlikely in the first two years. Therefore, the first year following AKI_OPT_ is a critical period for clinicians to halt the accelerated progression before patients suffered from persistent uremic symptoms. If patients’ dialysis-free status can be maintained in the first two years following AKI_OPT_, the risk of progression to ESRD would be gradually faded due to better preserved kidney function during the event of AKI_OPT_ or the more pronounced recovery in kidney function after the event of AKI_OPT_. Our findings regarding the fully adjusted cross-sectional associations between selected clinical factors such as history of CVD and exposures to NSAID or contrast prior to the event of AKI_OPT_ provides useful information on risk markers for development of AKI_OPT_ in real-world practice (Fig. 4). However, more research must be conducted to discover new risk factors or effective prevention for AKI_OPT_.

This study has several limitations. First, the Health and Welfare Data Science Center (HWDC) of Taiwan did not release the biochemical data through the Health Insurance Medical Information Cloud Inquiry System until 2017; therefore, patients’ serum creatinine measurements outside of our hospital were unavailable. Information bias could not be completely excluded; however, a high retention rate among patients in our hospital, which is the largest tertiary medical center in central Taiwan, should have effectively minimized the risk of misclassification. Second, we could not completely exclude the possibility of residual confounding and over-adjustment for variables that could be in the causal pathway. For example, detailed information on environmental factors such as diet, exposure to nephrotoxicants, and physical activity was not available. Third, the study population that were drawn from a pre-ESRD program poses a limitation in terms of generalizability. However, our proposed diagnostic cutoffs for the percent change of serum creatinine and eGFR approximated the 75^th^ percentile of the overall distribution, which improves generalizability of the proposed AKI_OPT_ algorithm to patients with normal kidney function as within-day variability of serum creatinine above 30% is rarely observed in general population (submitted data) (Supplementary Fig. S2).

In conclusion, we validated an AKI_OPT_ algorithm in the CKD population by demonstrating that this classification could accurately predict the risks of progression to ESRD and all-cause mortality. Our study also revealed that the use of conventional RIFLE-based AKI criteria significantly underestimates the role of AKI in the general population. Despite the full recovery of eGFR declining slope after AKI event, the loss of kidney function is not likely recovered, which strengths the causal link between AKI and CKD progression.

## Methods

### Study population

In 2002, Taiwan’s National Health Insurance launched the Project of Integrated Care of CKD, initially targeting patients with an eGFR lower than 60 mL/min/1.73 m^2^; since 2007, the program has used a multidisciplinary approach to focus on CKD stages 3b–5^34^. This pre-end-stage renal disease (ESRD) program utilizes a multidisciplinary approach (involving nephrologists, renal nursing specialists, pharmacists, healthcare educators and dieticians) to design individualized care plans for a wide range of CKD patients. China Medical University Hospital (CMUH) joined pre-ESRD program in 2003 and has been consecutively enrolling patients who were willing to participate this care program and had a diagnosis of CKD based either on the working diagnoses of nephrologists or in accordance with the criteria outlined in the National Kidney Foundation (NKF)/KDOQI Guidelines^35^. Up-to-date, the CMUH pre-ESRD program includes more than 11 000 participants with an overall retention rate of 90%. Patients in CKD stage 3b, 4 and 5 were, respectively, followed up at 12, 8 and 4 weeks, or when necessary. Biochemical markers of renal injury including serum creatinine, eGFR, and the spot urine protein–creatinine ratio (PCR) were measured at intervals of no more than 12 weeks^36^. All patients enrolled in the program were followed up until the initiation of maintenance dialysis for ESRD, loss to follow-up, death, or December 31, 2015. ESRD status was verified through active contact and review of electronic medical records (EMRs). Complete mortality data were obtained from the National Death Registry.

In this study, 6,046 patients aged 20–90 years who remained dialysis-naïve and had records for at least two eGFR measurements before pre-ESRD program enrollment were selected from among 10,277 program participants (the selection process is detailed in Supplementary Fig. S3). The index date was defined as the day of the first AKI event based on our proposed diagnostic criteria of the AKI_OPT_. All methods in this study were performed in accordance with the relevant guidelines/regulations. The study was approved by the Big Data Center of China Medical University Hospital and the Research Ethical Committee/Institutional Review Board of China Medical University Hospital (CMUH105-REC3-068) and the need to obtain informed consent for the present study was waived by the Research Ethical Committee of China Medical University Hospital.

### Criteria for outpatient acute kidney injury

We tracked all serum creatinine measurements of the patients up to 180 days before pre-ESRD enrollment. Serum creatinine was measured using the Jaffe rate method (kinetic alkaline picrate) at CMUH Central Laboratory using a Beckman UniCel DxC 800 immunoassay system (Beckman Coulter Inc., Brea, CA, USA). Calculations of eGFR were performed using the Chronic Kidney Disease Epidemiology Collaboration creatinine equation^37^. An AKI_OPT_ event was defined as a fluctuation of >50% in serum creatinine or >35% in eGFR in the 180-day period preceding pre-ERSD enrollment. The 180-day time frame was chosen based on prior evidence and represents the potentially longer duration of kidney vulnerability to nephrotoxins in elder patients or patients with CKD, particularly nonsteroidal anti-inflammatory drugs (NSAID)^38,39^. Baseline serum creatinine was defined as the best (lowest) serum creatinine within 180 days prior to the pre-ESRD enrollment. Fluctuations in serum creatinine were calculated as the difference between the maximum and minimum values of serum creatinine divided by the minimum value of serum creatinine. Fluctuations in eGFR were calculated as the difference between the maximum and minimum values of eGFR divided by the maximum value of eGFR. We further classified the AKI episode as deteriorating or stable based on the difference between the last S serum creatinine value in the 180-day period and baseline serum creatinine at the time of pre-ESRD enrollment. If the difference was positive and greater than 0.3 mg/dL, then the AKI_OPT_ was defined as deteriorating AKI, whereas if the difference was less than 0.3 mg/dL, it was categorized as stable AKI_OPT_. This cutoff was selected empirically based on KDIGO serum creatinine criteria for Stage 1 AKI^11^. An alternative definition using 0 mg/dL as the cut-off was also evaluated (Supplementary Table S3). Detailed information of other variables such as sociodemographic characteristics was provided in Supplementary text.

### Statistical analyses

Continuous variables are expressed as medians and interquartile ranges (IQRs) and were compared using the nonparametric Kruskal–Wallis test, whereas categorical variables are expressed as a frequency (percentage) and were compared using the chi-square test. Associations between AKI status (with and without AKI_OPT_, stable AKI_OPT_, and deteriorating AKI_OPT_) and the 1-year and overall risks of ESRD requiring dialysis and all-cause mortality were estimated using a multivariable Cox regression analysis. Multivariable Cox regression models were initially adjusted for sociodemographic and lifestyle variables, including age, sex, education (<9, 9–12, 12–16 or >16 years), smoking status (never, former or current) and alcohol consumption (never, former or current), followed by adjustments for comorbidities including diabetes mellitus, hypertension, CVD, primary etiologies of CKD, baseline medications (details provided in Table 1) and the baseline serum creatinine defined by the best (lowest) serum creatinine identified within the diagnostic window of AKI_OPT_. For outcomes of progression to ESRD, we performed competing risk analysis in accordance with the methods outlined by Fine and Gray to minimize potential bias introduced by a competing risk of death^40^. We also applied coarsened exact matching (CEM) analysis with matching criteria of age, sex, baseline eGFR, diabetes, hypertension, and CVD to specifically adjust for imbalances in baseline kidney function between patients with and without AKI_OPT_^41^.

To compare the eGFR progression change before and after the index episode AKI_OPT_, we further identified a total of 1,106 patients who had undergone at least three serum creatinine measurements within a 2-year interval before and after the index AKI_OPT_ event. We applied the growth piecewise linear mixed model by incorporating random effects for correlated eGFR measurements on the same patient to understand the effect of AKI_OPT_ events on CKD progression using the following equation^42^:

$$eGF{R}_{ij}={\beta }_{0}+{\beta }_{1}(Ag{e}_{ij}-Ag{e}_{atAKI})\times {\delta }_{ij}+{\beta }_{2}(Ag{e}_{ij}-Ag{e}_{atAKI})\times (1-{\delta }_{ij})+{\varepsilon }_{ij}$$

where *δ*_*ij*_ = 1 for the period before the AKI_OPT_ event and *δ*_*ij*_ = 0 for the period after the AKI_OPT_ event.

Lastly, we used a multivariable logistic regression model to investigate the risk markers, such as demographic and selected clinical factors, for developing AKI_OPT_. All statistical analyses were performed in SAS (version 9.4, SAS Institute Inc., Cary, NC, USA) and R (version 3.2.3, R Foundation for Statistical Computing, Vienna, Austria). The 2-sided statistical significance level was set at α = 0.05.

### Ethical approval

The study was approved by the Research Ethical Committee/Institutional Review Board of China Medical University Hospital (CMUH105-REC3-068).

## Supplementary information

Supplementary material

## Data Availability

The data that support the findings of this study are available on request from the corresponding author, CCK. The data are not publicly available due to them containing information that could compromise research participant privacy.
