# Supplementary material for: Acute Kidney Injury in the Outpatient Setting Associates with Risk of End-Stage Renal Disease and Death in Patients with CKD
Source: Sci Rep. 2019 Nov 27;9:17658. doi: 10.1038/s41598-019-54227-6 (PMC6881443; doi:10.1038/s41598-019-54227-6)
Supplement: Supplementary file 1 — Supplementary material [file 41598_2019_54227_MOESM1_ESM.docx]

**Supplementary Material**

**Acute Kidney Injury in the Outpatient Setting Associates with Risk of End-Stage Renal Disease and Death in Patients with CKD**

Hung-Chieh Yeh^1,2^, I-Wen Ting^1,2^, Han-Chun Huang^3^, Hsiu-Yin Chiang^3^, Chin-Chi Kuo^1-4^

^1^ AKI-CARE (Clinical Advancement, Research and Education) Center, Department of Internal Medicine, China Medical University Hospital and College of Medicine, China Medical University, Taichung, Taiwan

^2^ Division of Nephrology, Department of Internal Medicine, China Medical University Hospital and College of Medicine, China Medical University, Taichung, Taiwan

^3^ Big Data Center, China Medical University Hospital and College of Medicine, China Medical University, Taichung, Taiwan

^4^ School of Medicine, College of Medicine, China Medical University, Taichung, Taiwan

**Corresponding author:** Chin-Chi Kuo, MD, PhD.

School of Medicine, College of Medicine, China Medical University, Taichung, Taiwan; Big Data Center, China Medical University Hospital and College of Medicine, China Medical University, Taichung, Taiwan

**Address:** 2, Yude Rd., North Dist., Taichung City 404

**Tel:** 886.4.2205.2121-2910

**Email:** chinchik@gmail.com

**Supplementary text**

**Table S1.** Estimates of the main fixed effects obtained from the growth piecewise mixed-effects modeling stratified by renin-angiotensin inhibitors.

**Table S2**. Baseline characteristic between patients without AKI_OPT_ and with AKI_OPT_ after coarsened exact matching.

**Table S3.** Hazard ratios (95% confidence interval) for risk of progression to ESRD and all-cause mortality by the presence of preceding AKI_OPT_ using different definition to define stable and deteriorating AKI_OPT_.

**Figure S1.** eGFR slope (red line) with the light red shaded area representing 95% confidence intervals before and after the AKI_OPT_ event, stratified by the use of renin-angiotensin inhibitors (ACEIs and ARBs) within 90 days prior to the event of AKI_OPT_. Blue and orange points represent eGFR measurements before and after enrollment into pre-ESRD program. ACEI: angiotensin-converting enzyme inhibitor; ARB: angiotensin II receptor blocker.

**Figure S2.** Box-percentile plot of the percent change of the serum creatinine and estimated glomerular filtration rate (eGFR) levels in overall study population.

**Figure S3.** Flow diagram of patient selection

**Supplementary Text**

***Other variables***

Sociodemographic variables collected during the enrollment interview included age, sex, education, cigarette smoking status, and alcohol consumption. Smoking status and alcohol consumption were categorized as never, former, and current. Body mass index (BMI) was calculated as weight in kilograms divided by height in square meters. Diabetes mellitus and hypertension were defined using physicians’ clinical diagnoses according to ICD-9-CM codes and the use of glucose-lowering or antihypertensive agents. History of cardiovascular disease (CVD) was defined as a record of coronary artery disease, myocardial infarction, stroke, or heart failure in EMRs. Baseline comorbidities, medication use, and relevant biochemical measures were determined according to registry data or information obtained from the EMRs within a 1-year window prior to enrollment.

**Table S1.** Estimates of the main fixed effects obtained from the growth piecewise mixed-effects modeling stratified by renin-angiotensin inhibitors.

|  | **Growth piecewise mixed-effects model** | | | | |
| --- | --- | --- | --- | --- | --- |
|  | **Estimate ± SE**  **(ml/min/1.73m^2^)** | ***p*-value** |  | **Estimate ± SE**  **(ml/min/1.73m^2^)** | ***p* -value** |
|  | **Non renin-angiotensin inhibitors** | |  | **Renin-angiotensin inhibitors** | |
| Intercept | 25.13 **±** 0.92 | 0.031 |  | 24.46 ± 0.76 | 0.324 |
| Pre-AKI_OPT_ slope (yr^-1^) | -10.68 **±** 0.50 | <0.001 |  | -10.55 ± 0.42 | <0.001 |
| Post-AKI_OPT_ slope (yr^-1^) | 1.01 **±** 0.47 | <0.001 |  | -0.39 ± 0.40 | <0.001 |

$$\mathbf{Linear model}{: eGFR}_{ij}={}_{0}+{}_{1}\left( {Age}_{ij}-{Age}_{at AKI} \right)\times{}_{ij}+ {}_{2}\left( {Age}_{ij}-{Age}_{at AKI} \right)\times\left( 1-{}_{ij} \right)+ \varepsilon_{ij}$$

${}_{ij}$=1, for the time period before AKI event; ${}_{ij}$=0, for the time period after AKI event.

**Abbreviations:** AKI_OPT_, acute kidney injury in outpatient setting; SE, standard error.

**Table S2**. Baseline characteristic between patients without AKI_OPT_ and with AKI_OPT_ after coarsened exact matching.

|  | **No AKI_OPT_** | **AKI_OPT_** | ***p*-value** |
| --- | --- | --- | --- |
| **n** | 1600 | 1600 |  |
| Age at entry (year) | 69.33 (59.67, 76.93) | 69.43 (59.72, 77.20) | 0.850 |
| Woman, n (%) | 741 (46.31) | 741 (46.31) | 1.000 |
| Diabetes, n (%) | 811 (50.69) | 811 (50.69) | 1.000 |
| Hypertension, n (%) | 1104 (69.00) | 1104 (69.00) | 1.000 |
| Cardiovascular disease, n (%) | 746 (46.63) | 746 (46.63) | 1.000 |
| eGFR (mL/min/1.73m^2^) | 20.54 (10.60, 32.97) | 20.07 (10.69, 32.80) | 0.613 |

*p*-values are calculated by Wilcoxon rank sum test for continuous variables and chi-square test for categorical variables

Multivariate L1 distance of matched population: L1=0.539.

**Abbreviations:** AKI_OPT_, acute kidney injury in outpatient setting; eGFR, estimated glomerular filtration rat

**Table S3.** Hazard ratios (95% confidence interval) for risk of progression to ESRD and all-cause mortality by the presence of preceding AKI_OPT_ using different definition to define stable and deteriorating AKI_OPT_.

|  |  |  | **Original definition for stable and deteriorating AKI_OPT_** |  |  |  | **Alternative definition for stable and deteriorating AKI_OPT_** |
| --- | --- | --- | --- | --- | --- | --- | --- |
|  | **N** | **cases** | **Adjusted HR (95% CI)** |  | **N** | **cases** | **Adjusted HR (95% CI)** |
| **ESRD requiring dialysis**† |  |  |  |  |  |  |  |
| **1-year dialysis** |  |  |  |  |  |  |  |
| No AKI_OPT_ | 4141 | 321 | 1.00 (Ref) |  | 4141 | 321 | 1.00 (ref) |
| Stable AKI_OPT_ | 1537 | 176 | 1.33 (1.09, 1.62) |  | 550 | 96 | 2.08 (1.63, 2.64) |
| Deteriorating AKI_OPT_ | 368 | 110 | 3.71 (2.93, 4.69) |  | 1355 | 190 | 1.66 (1.37, 2.01) |
| **Overall** |  |  |  |  |  |  |  |
| No AKI_OPT_ | 4141 | 753 | 1.00 (Ref) |  | 4141 | 753 | 1.00 (ref) |
| Stable AKI_OPT_ | 1537 | 314 | 1.24 (1.07, 1.43) |  | 550 | 172 | 1.79 (1.50, 2.13) |
| Deteriorating AKI_OPT_ | 368 | 146 | 2.68 (2.17, 3.31) |  | 1355 | 288 | 1.37 (1.18, 1.59) |
| **All-cause mortality** |  |  |  |  |  |  |  |
| **1-year mortality** |  |  |  |  |  |  |  |
| No AKI_OPT_ | 4141 | 131 | 1.00 (Ref) |  | 4141 | 131 | 1.00 (ref) |
| Stable AKI_OPT_ | 1537 | 130 | 2.14 (1.66, 2.77) |  | 550 | 56 | 2.70 (1.95, 3.73) |
| Deteriorating AKI_OPT_ | 368 | 26 | 2.56 (1.67, 3.92) |  | 1355 | 100 | 2.00 (1.53, 2.63) |
| **Overall** |  |  |  |  |  |  |  |
| No AKI_OPT_ | 4141 | 377 | 1.00 (Ref) |  | 4141 | 377 | 1.00 (ref) |
| Stable AKI_OPT_ | 1537 | 238 | 1.66 (1.40, 1.98) |  | 550 | 103 | 1.84 (1.47, 2.31) |
| Deteriorating AKI_OPT_ | 368 | 49 | 2.23 (1.64, 3.02) |  | 1355 | 184 | 1.69 (1.40, 2.04) |

**Original definition:** If the difference between the last S-Cre value in the 180-day period and baseline S-Cre at the time of pre-ESRD enrollment was larger than 0.3 mg/dL, the AKI_OPT_ was defined as deteriorating AKI_OPT_ whereas if the difference was less than 0.3 mg/dL, it was stable AKI_OPT_

**Alternative definition:** If the difference was positive (difference of S-Cr > 0), the AKI_OPT_ was defined as deteriorating AKI_OPT_ whereas if the difference of S-Cre was negative (difference of S-Cr ≤ 0), it was stable AKI_OPT_

† With competing risk analysis for death

Models were adjusted for age at entry, gender, smoking status, alcohol consumption, education, diabetes, hypertension, cardiovascular disease, primary etiologies of CKD, and medication utilization within a 90-day period prior to AKI_OPT_ (n=6024).

**Abbreviations:** AKI_OPT_, acute kidney injury in outpatient setting; ESRD, end-stage renal disease; CI, confidence interval; HR, hazard ratio.

**Figure S1.** eGFR slope (red line) with the light red shaded area representing 95% confidence intervals before and after the AKI_OPT_ event, stratified by the use of renin-angiotensin inhibitors (ACEIs and ARBs) within 90 days prior to the event of AKI_OPT_. Blue and orange points represent eGFR measurements before and after enrollment into pre-ESRD program. ACEI: angiotensin-converting enzyme inhibitor; ARB: angiotensin II receptor blocker.


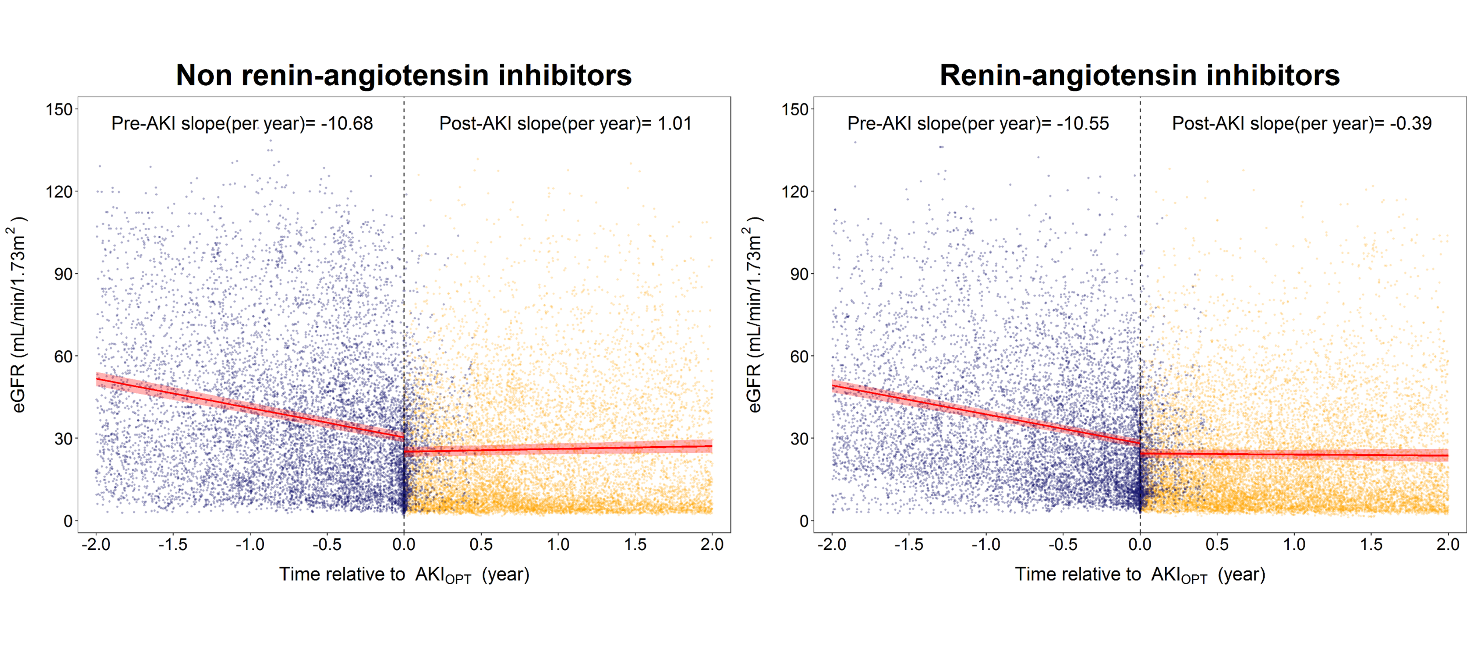


**Figure S2.** Box-percentile plot of the percent change of the serum creatinine and estimated glomerular filtration rate (eGFR) levels in overall study population.


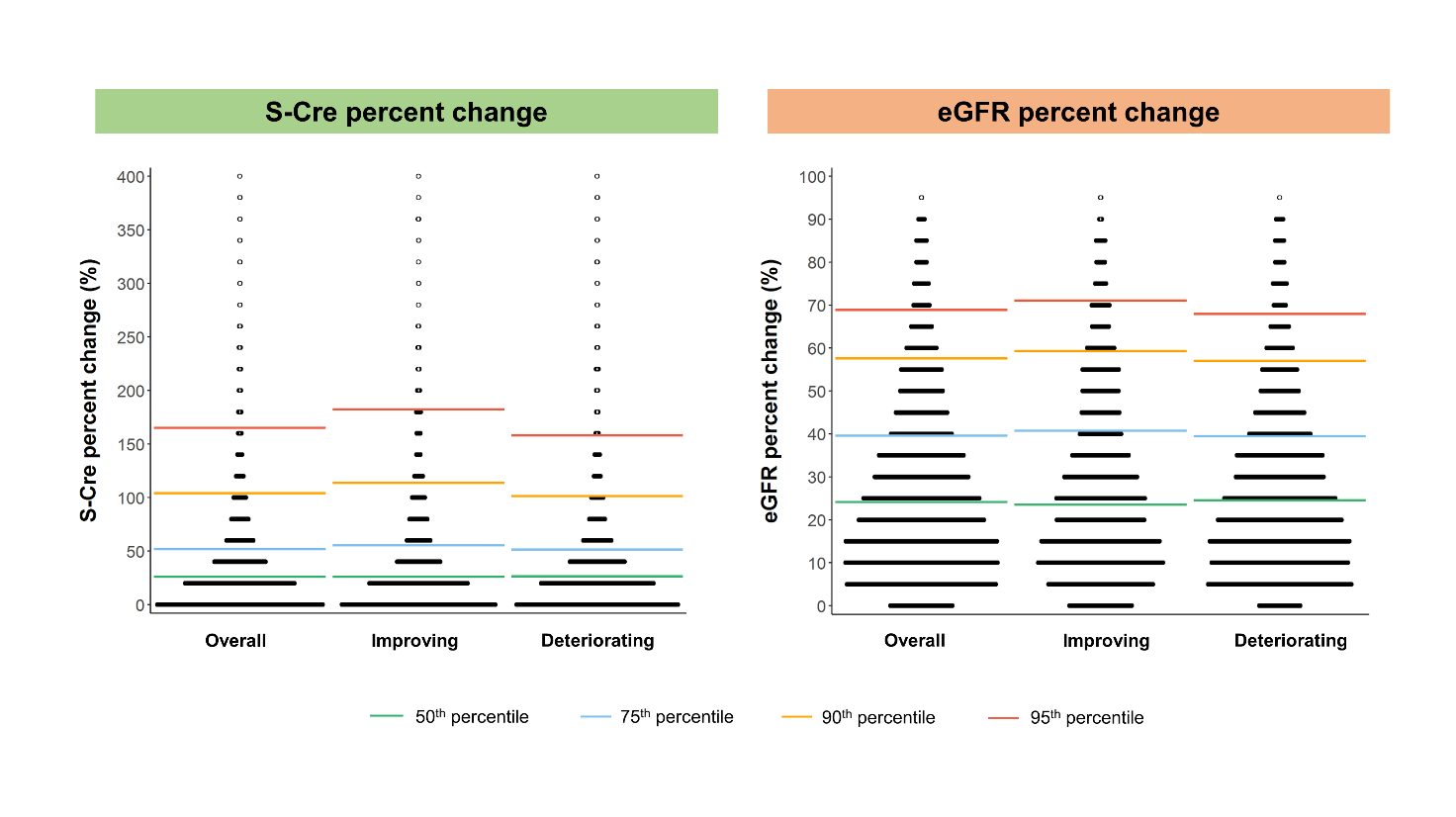


**Figure S3.** Flow diagram of patient selection

**
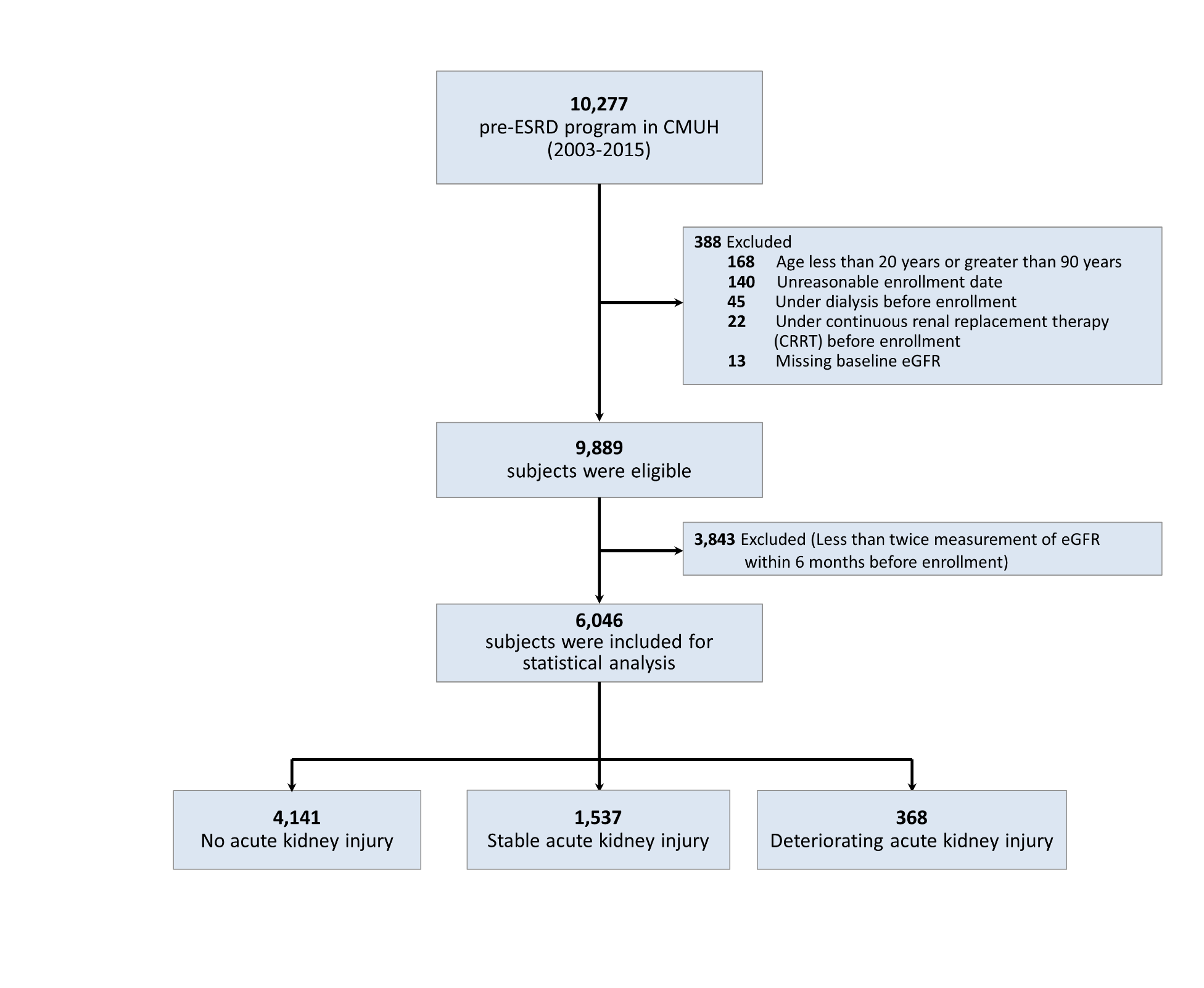
**
